# Coping strategies used by migrant women during pregnancy. An integrative review

**DOI:** 10.17533/udea.iee.v43n3e10

**Published:** 2025-11-06

**Authors:** Ivett Adriana Herrera Zuleta, Angélica María Ospina Romero, Abraham Isaac Esquivel Rubio, Claudia Jennifer Domínguez Chavez

**Affiliations:** 2 Nurse, Ph.D. Professor. Email: angelica.ospina@unisabana.edu.co Universidad de la Sabana Colombia angelica.ospina@unisabana.edu.co; 3 Nurse, Ph.D. Professor. Email: abraham.esquivel@uabc.edu.mx Universidad Autónoma de Baja California Mexico abraham.esquivel@uabc.edu.mx; 4 Nurse, Ph.D. Professor. Email: jennifer.dominguez@uabc.edu.mx Universidad Autónoma de Baja California Mexico jennifer.dominguez@uabc.edu.mx; 5 Universidad de la Sabana. Chía, Colombia Universidad de la Sabana Universidad de la Sabana Chía Colombia; 6 Universidad Autónoma de Baja California. Mexicali, México. Universidad Autónoma de Baja California Universidad Autónoma de Baja California Mexicali Mexico

**Keywords:** pregnancy, emigrants and immigrants, psychological adaptation., embarazo, emigrantes e inmigrantes, adaptación psicológica, gravidez, emigrantes e imigrantes, adaptação psicológica.

## Abstract

**Objective.:**

To analyze the coping strategies for the adaptation of the migrant during pregnancy described in the literature.

**Methods.:**

Integrative review using the method approached by Whittemore and Knafl. MeSH terms Pregnancy, Migrants and immigrants, and Psychological adaptation, were incorporated in addition to their variants in the databases of Pubmed, Scopus, EBSCO, Science Direct, and Web of Science. Articles that examined the coping strategies used by pregnant migrants to adapt during pregnancy were included. Only the articles published from 2003 to 2023 was considered. After applying the inclusion and exclusion criteria, the articles were analyzed with the CASPe critical reading tool in which quality and consistency were reviewed. Using the software, Atlas.ti, version 23.2.1, content analysis for the categorical construction of data was performed. These strategies were analyzed with the middle-range theory adaptation to life events by Callista Roy.

**Results.:**

. A total of 416 articles were considered. The reviewed articles show 14 coping strategies used by the pregnant migrant, which were grouped into 5 strategies called social support, emotional regulation and transfer, positive attitude strategy, cultural adaptation strategy, and comprehensible language strategy.

**Conclusion.:**

It was found that the strategy most used by the migrant pregnant woman is social support, followed by positive attitude strategies and comprehensible language; when using these strategies, the results show migrant pregnant women with a decrease in anxiety, fear and a positive attitude towards life and health.

## Resumen

**Objetivo.:**

Analizar las estrategias de afrontamiento para la adaptación de la migrante durante la gestación descritas en la literatura**.**

**Métodos.:**

Revisión integradora utilizando el método propuesto por Whittemore y Knafl. Se incorporaron para la búsqueda los términos MeSH de: Embarazo; Emigrantes e inmigrantes; y Adaptación psicológica; además de sus variantes en las bases de datos Pubmed, Scopus, EBSCO, Science Direct y Web of Science. Se incluyeron artículos que examinaban las estrategias de afrontamiento utilizadas por las migrantes embarazadas para adaptarse durante la gestación. La revisión incluyó los artículos publicados de 2003 a 2023, en idiomas inglés, español y portugués. A los artículos recuperados se les revisó la calidad y la coherencia con la herramienta de lectura crítica CASPe. Se utilizó el software Atlas.ti, versión 23.2.1. para el análisis de contenido necesario para la construcción categórica de los datos con la teoría de alcance medio «adaptación a los acontecimientos de la vida» de Callista Roy**.**

**Resultados.:**

Se consideraron un total de 416 artículos. Tras su revisión, 17 artículos mostraban 14 experiencias de estrategias de afrontamiento utilizadas por las migrantes embarazadas, las cuales se agruparon en cinco categorías denominadas: apoyo social, regulación y transferencia emocional, actitud positiva, adaptación cultural, y lenguaje comprensible**.**

**Conclusión.:**

Las mujeres migrantes durante la gestación utilizan estrategias de afrontamiento para la disminución de la ansiedad y el miedo, y adoptan una actitud positiva hacia la vida y la salud. En este sentido, es necesario fortalecer en el personal de salud la competencia cultural ante los fenómenos migratorios con el fin de para garantizar el cuidado humano, equitativo y de alta calidad en la materna.

## Resumo

**Objetivo.:**

analisar as estratégias de enfrentamento descritas na literatura para a adaptação de mulheres migrantes durante a gravidez.

**Métodos.:**

revisão integrativa utilizando o método proposto por Whittemore e Knafl. Os termos MeSH: gravidez; migrantes e Imigrantes; e adaptação psicológica foram incorporados à busca, juntamente com suas variantes, nas bases de dados Pubmed, Scopus, EBSCO, Science Direct e Web of Science. Foram incluídos artigos que examinavam as estratégias de enfrentamento utilizadas por migrantes grávidas para se adaptarem durante a gravidez. A revisão incluiu artigos publicados de 2003 a 2023, em inglês, espanhol e português. Os artigos recuperados foram revisados quanto à qualidade e consistência utilizando a ferramenta de leitura crítica CASPe. O software Atlas.ti, versão 23.2.1, foi utilizado para a análise de conteúdo necessária à construção categorial dos dados, utilizando a teoria de médio alcance de Callista Roy, "adaptação aos eventos da vida".

**Resultados.:**

Foram considerados um total de 416 artigos. Após a revisão, 17 artigos apresentaram 14 experiências de estratégias de enfrentamento utilizadas por gestantes migrantes, que foram agrupadas em cinco categorias: apoio social, regulação emocional e transferência, atitude positiva, adaptação cultural e linguagem compreensível.

**Conclusão.:**

Mulheres migrantes durante a gravidez utilizam estratégias de enfrentamento para reduzir a ansiedade e o medo e adotar uma atitude positiva em relação à vida e à saúde. Nesse sentido, é necessário fortalecer a competência cultural dos profissionais de saúde em relação aos fenômenos migratórios, a fim de garantir um atendimento humano, equitativo e de alta qualidade na maternidade.

## Introduction

The mobility of people between countries is permanent and sometimes occurs unexpectedly. Some causes are: climate change; the search for quality of life; economic, political, social conflicts; and gender violence, which have been considered determinants for more women to carry out the migratory process.^1^^,^^2^ According to the International Organization for Migration (IOM), women represent 48% of people who migrate, which has been increasing in recent decades; especially for those who do it alone, being pregnant or the head of the household, care becomes a challenge for health services.^3^^,^^4^ The pregnancy of women who migrate, their physical, psychological and social condition, the lack of accessibility to healthcare, the deficit in prenatal care, and the growing maternal and neonatal morbidity and mortality place them in a vulnerable condition.^5^ According to the United Nations (UN) report, migration is linked to structural transformations in the lives of Migrant Pregnant Women (MPW), thus leading them to generate strategies to deal with the changes that occur during pregnancy and migration.^6^

A theoretical contribution that has attempted to explain the processes of coping and adaptation to life events, such as pregnancy and unexpected situations such as migration, is that described by Callista Roy, with the middle-range theory of “Adaptation to life events”. Roy defines the process of adaptation as the ways in which a person responds to changes in the environment, based on patterns of responses that lead to the use of coping styles and strategies to effectively adapt to situational health challenges such as pregnancy. If coping strategies are effective, they lead to positive adaptation to the environment.^7^ Coping strategies can be innate or acquired by the individual, with which he/she responds to internal and external demands that arise from changes in the environment such as during migration.^8^ In the middle-range theory of situational events proposed by Roy, nursing practice aims to promote people's adaptation by assessing factors that influence adaptive capacity to support environmental management and promote coping.^9^^,^^10^

No evidence was found in the literature of the application of the theory of adaptation to life events in migrant pregnant women. Therefore, it is necessary to find what are the coping strategies are used in this population and thus, the following research question is posed: ¿What are the coping strategies for the adaptation of migrant pregnant women described in the literature? 

## Methods

**Figure 1:**
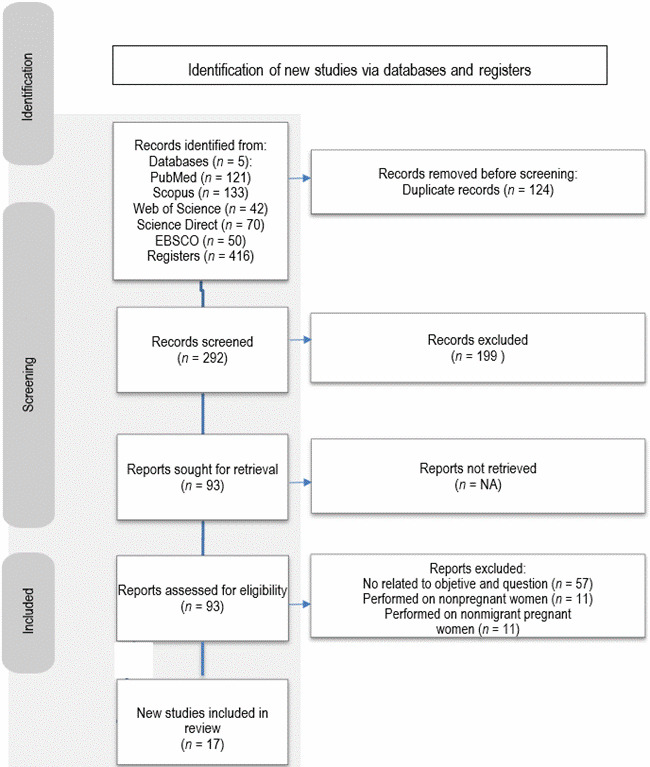
Flow Chart of the article selection process

Integrative literature review taking into account the steps proposed by Whittemore and Knafl ^11^ complying with the methodological rigor with the following five stages: 

(i) Identification of the problem, in which the description of the phenomenon to be investigated described by the coping strategies used by the pregnant migrants was carried out. 

(ii) The literature search was carried out in 5 databases (Pubmed, Scopus, EBSCO, Science direct, and the Web of Science platform) to find the coping strategies MPW develop to adapt to the migration process. Studies with any type of methodology was accepted. Publications from 2003 to 2023 were considered given the first approaches to the topic for this population; for the search strategy, the following keywords were used: Pregnancy, Emigrants and Immigrants, and Psychological Adaptation, included in the Descriptors in Health Sciences (DeCS) and the Medical Subject Headings (MeSH), as well as synonyms, related terms, spelling variations and abbreviations. The Boolean operators AND OR were incorporated for the search. All research designs, written in English, Spanish and Portuguese were considered. An example of a complete search strategy is: N1 ENT#091;((((((((Immigrants and Emigrants) OR (Immigrants)) OR (Immigrant)) OR (Foreigners)) OR (Foreigner)) OR (Aliens)) OR (Alien)) OR (Emigrants)) OR (Emigrant)ENT#093; AND N2 ENT#091;((((((((((((Pregnancy) OR (Pregnant)) OR (Maternal)) OR (Maternity)) OR (Midwifery)) OR (Birth)) OR (Perinatal)) OR (Intrapartum)) OR (Antenatal)) OR (Postnatal)) OR (Childbearing)) OR (Prenatal)) OR (Motherhood)ENT#093; and N3 ENT#091;(((((((((((((((((Adaptation, Psychological) OR (Adaptation, Psychologic)) OR (Psychologic Adaptation)) OR (Psychological Adaptation)) OR (Adjustment)) OR (Coping Behavior)) OR (Behavior, Coping)) OR (Behaviors, Coping)) OR (Coping Behaviors)) OR (Coping Skills)) OR (Coping Skill)) OR (Skill, Coping)) OR (Coping Strategies)) OR (Strategies, Coping)) OR (Behavior, Adaptive)) OR (Adaptive Behavior)) OR (Adaptive Behaviors)) OR (Behaviors, Adaptive)ENT#093;. Articles examining MPW’S coping strategies used for adaptation during pregnancy were included. The exclusion criteria were studies that were outside the topic of interest, that is, with non-pregnant migrant women and studies with internal migrants (urban to rural area of the same country where they were carried out). 

(iii) Data evaluation, after the search for articles, they were moved to the Rayyan QCRI platform^®^ (the Systematic Reviews web application) for analysis. A total of 416 studies were recovered, assessed, and selected for relevance for inclusion based on the information provided in the title and abstract. After using Rayyan application, 124 duplicate articles were removed; two authors made the selection simultaneously and disagreements about the inclusion of studies were solved through discussion with a third researcher (Figure 1). 

(iv) Data analysis, the methodological guidelines of the Critical Appraisal Skills Programme (CASPe) were used to analyze the data quality of the articles, taking into account the type of study design, methodological consistency, quantitative methodological rigor (validity and reliability) and qualitative rigor (confirmability, credibility), coherence between the elements of the study and the relevance of the phenomenon.^12^ A matrix was made in Microsoft Excel, for analysis of each article in which data were extracted related to authors, year, country, title, purpose of the study, study design, conclusions, the main findings of the study, the coping strategy used by the pregnant migrant woman, and the result obtained when using the strategy. These results were analyzed in a data extraction form for subsequent analysis and synthesis. 

(v) The results are presented below.

To categorize the information, the content analysis technique was used by means of the qualitative information analysis program Atlas.ti, version 23.2.1, which made it possible to simplify and compile the information from 17 articles in an analysis matrix with which categories were generated, which were related to the coping strategies proposed by the mid-range theory of adaptation to life events proposed by Callista Roy.^13^

## Results

As shown in Table 1, seventeen articles were reviewed that documented the coping strategies used by MPW for adaptation during pregnancy; 9 studies were with qualitative design, 3 with quantitative analytical designs, 1 mixed study, 2 integrative reviews, and 2 systematic reviews. Of these, 9 were carried out on the American continent from 2004 to 2023; 4 in Europe, from 2015 to 2023; 1 in Oceania in 2019 and 3 in Asia in 2012, 2014 and 2016. The articles identified 14 coping strategies that migrants used in the form of skills and tools to maintain health during pregnancy.^14^

**Table 1:** Description of articles Selected for Analysis

Author(s) / year of publication/ country / Citatios	Objective of study	Title	Methods	Conclusions
Sibrian N. 2021 / Chile.^15^	To identify the role of emotions in the trajectory of a pregnant Venezuelan user of the Chile Crece Contigo program.	Emotional adjustments of a pregnant woman and immigrant in Chile: strategies to reduce suffering.	Case study, with biographical approach and ethnographic perspective.	Patience in the face of waiting, laughter in the face of pain and gratitude in the face of a right become adaptation strategies of a social group facing a certain type of hospitality. Generally, all hospitality is conditional and is almost never free of debt. But, if we add to this social class, skin color, country of origin or political orientation of the guest, these characteristics could be additional arguments for hospitality to become a strongly demarcated boundary. Strategies of emotional regulation or transference would be a reflection not only of ways of coping with suffering, but also the product of a differentiated mode of insertion of migrants.
Song JE, *et al.,* / 2016 / Korea.^16^	To synthesize the evidence of immigrant women's experiences of maternal adaptation in Korea.	A qualitative review of immigrant women's experiences of maternal adaptation in South Korea.	A qualitative systematic review was performed by means of thematic synthesis.	These findings demonstrate the importance of understanding and improving maternal adaptation of immigrant women living in Korea. This can be achieved by enhancing social support, providing culturally sensitive maternal healthcare services, and expanding opportunities for immigrant women in education, job training, and economic independence.
Higginbottom GMA, *et al.,* / 2014 / Canada.^17^	To synthesize data on immigrant women's experiences of maternity services in Canada.	Immigrant women's experience of maternity services in Canada: A meta-ethnography.	A qualitative systematic literature review using a meta-ethnographic approach.	In order to implement woman centered care, to enhance access to maternity services, and to promote immigrant women's health, it is important to consider these women's social position, cultural knowledge and beliefs, and traditional customs in the health care.
Lukin TT, *et al.,* / 2023 / Sweden.^18^	To describe Syrian women’s experiences of being pregnant and receiving care at antenatal clinics for the first time after migration.	Syrian women’s experiences of being pregnant and receiving care at antenatal clinics in Sweden for the first time after migration.	A phenomenological lifeworld approach was used.	Syrian women’s experiences reveal a heterogeneous group with different experiences and background. The study highlights the first visit and emphasizes the importance of this visit for future quality of care. It also points out the negative occurrence of the transferring guilt from the midwife to the migrant woman in case of cultural insensitivity and clashing norm systems.
Lin ML, Wang HH./ 2008 /T aiwan.^19^	To examine the relationships between the knowledge of pregnancy, attitude toward pregnancy and experience of medical services, and prenatal examination behavior of pregnant Southeast Asian women in Taiwan.	Prenatal examination behavior of Southeast Asian pregnant women in Taiwan: A questionnaire survey	This was a cross-sectional study with a structured questionnaire administered to participants.	The attitude toward childbearing of the participants was significantly correlated with their prenatal examination behavior. They require professional help in seeking out appropriate medical services that will improve their healthcare quality during pregnancy.
Viken B, *et al.,* / 201 5/ Norway.^20^	To explore the maternal health coping strategies of migrant women in Norway. The ethnic and cultural. background of the Norwegian population have become increasingly diverse	Maternal health coping strategies of migrant women in Norway	In this study a qualitative exploratory, descriptive design with a hermeneutic approach was employed.	To provide quality care, healthcare professionals should focus on the development of migrant women’s capabilities. Adaptation of maternal health services for culturally diverse migrant women also requires a culturally sensitive approach on the part of healthcare professionals.
Kim P./ 2020 / United States.^21^	Review of emerging literature on the role of stress in brain adaptation of human mothers during the perinatal period.	How stress can influence brain adaptations to motherhood.	Literature review.	I have reviewed available evidence that exposure to different types of stress, from childhood to the perinatal period, is associated with disrupted brain adaptation to motherhood, which can further increase risks for difficulties in developing sensitive parenting behaviors among new mothers.
González-Mesa E, *et al.,* / 2018 / Spain.^22^	To determine the influence of social and cultural factors on the mood state of a multicultural sample of 514 Turkish and Spanish pregnant women at the beginning of the pregnancy.	Cultural factors influencing antenatal depression: A cross-sectional study in a cohort of Turkish and Spanish women at the beginning of the pregnancy	Quantitative study designs. Cohort Study.	Our results confirm the existence of important differences in prevalence between Turkish (30.0%) and Spanish (9.9%) pregnant women. Some sociocultural features like having more children, unplanned pregnancies, or perceiving poor support from the partner, become important vulnerability factors.
Qian Y, Mao Y / 202 1/ United States.^23^	Explores how Chinese immigrant mothers use the ethnic social media-WeChat to engage in health information sharing and coping with cultural differences in healthcare between the U.S. and China.	Coping with cultural differences in healthcare: Chinese immigrant mothers’ health information sharing via WeChat.	Qualitative, integrative review, content analysis.	They adopt various acculturation strategies to manage the cultural differences in healthcare beliefs, practices, and systems.
Page RL / 2004 / United States.^24^	To provide an integrated review of the literature of potential explanations for better-than-expected pregnancy outcomes in Mexican immigrants, focusing on socioeconomics, social support, desirability of pregnancy, nutrition, substance use, religion, acculturation, and prenatal care.	Positive pregnancy outcomes in Mexican immigrants: what can we learn?	Literature was selected from refereed publications in the areas of nursing, medicine, public health, family, and sociology.	Low birth weight and prematurity are public health concerns in the United States. Through further study of the factors that lead to superior birth outcomes among Mexican immigrant women, rates of low birth weight and prematurity in the United States may be reduced.
Nguyen N / 2023 / United States. ^25^	This empirical research aims at shedding light on how such mothers use social media groups for social support seeking/providing regarding health utilization during their acculturation process.	Strangers helping strangers in a strange land: Vietnamese immigrant (expectant) mothers in the US use social media to navigate health issues in acculturation.	Drawing from Andersen’s Behavioral Model of Health Utilization, acculturation, and online social support conceptual frameworks, this study analyzes 18 in-depth interviews with immigrant Vietnamese (expectant) mothers in the United States on the use of social media in navigating health acculturation during their pregnancy and motherhood.	This research provides insights into personal experience on the uses of social media in navigating health behavior in the process of acculturation among Vietnamese immigrant (expectant) mothers in the United States. Their search seeks to contribute to the conceptual frameworks and practical experience of behavioral model of health utilization among immigrant Vietnamese ethnic immigrant pregnant women and mothers of babies and toddlers in navigating health during acculturation process in the United States. The limitations and future research suggestions are also discussed.
McLeish J, Redshaw M. / 2020 / England.^26^	To explore the experiences of disadvantaged mothers who received social support from a volunteer, through the lens of the theoretical framework of stress and coping and a multidimensional model of social support	She come like a sister to me’: a qualitative study of volunteer social support for disadvantaged women in the transition to motherhood in England.	This qualitative study in two stages was informed by the theoretical perspective of phenomenological social psychology.	Volunteer social support may have particular salience for mothers who lack structural support and need skilled functional support.
Qureshi R, Pacquiao DF / 2013 / Pakistan and United States.^27^	Describe the comparative birthing experiences of Pakistani immigrant women in Pakistan and the United States.	Ethnographic study of experiences of Pakistani women immigrants with pregnancy, birthing, and postpartum care in the United States and Pakistan.	Qualitative design using purposive sample of 26 women immigrants who originated from any province in Pakistan and experienced childbirth in Pakistan and in the United States.	The study has implications for designing culturally congruent and competent care for Pakistani women and their families. Planning and implementation of care must be informed by their own priorities on how valued practices should be accommodated. Involvement of social services and culturally congruent support network can facilitate meaningful access to health care services. Fostering link age between health care organizations and immigrant families can help design culturally competent services for this group.
Stanhope KK, *et al.,* / 2021 / United States.^28^	To examine how external stressors and coping strategies prior to and during pregnancy are reflected in Latina women's narratives about their lives through an Ecosocial framework.	Perceptions of stress and resilience among Latina women enrolled in prenatal care in Metro Atlanta through an ecosocial lens	This mixed methods research study explores pregnant Latina women's psychosocial wellbeing before and during pregnancy based on ecosocial theory.	The majority of women felt they should control emotional responses to external stressors during pregnancy to protect their baby's health. Women described motherhood and previous challenges as sources of maturity and improved coping. Familial financial and emotional support were perceived as critical to women's successful coping.
Knorr DA, Fox M. / 2023 / United States.^29^	We assessed social support, geographic proximity, and communication between the fetus’ grandmothers and pregnant mother.	An evolutionary perspective on the association between grandmother-mother relationships and maternal mental health among a cohort of pregnant Latina women.	Quantitative study designs. Cohort Study.	This study explores whether grandmother relationship characteristics are associated with prenatal mental health, motivated by the premise that positive associations are beneficial to the success of the pregnancy. Here, we suggest that grandmaternal allomothering includes the prenatal period. We observe that social support and communication with MGMs, but not PGMs, are associated with mental health benefits for mothers. More work is needed to connect this prenatal grandmaternal influence to offspring postnatal outcomes.
Quintanilha M, *et al.,* RC/2016/ Canada. ^30^	We explored migrant women’s perceptions and experiences of health during pregnancy and postpartum, while participating in a perinatal program offered through a community-based organization. Additionally, we examined sociocultural factors that might have shaped women’s health upon migration to the Canadian city of Edmonton, Alberta.	Contrasting “back home” and “here”: how Northeast African migrant women perceive and experience health during pregnancy and postpartum in Canada.	A community-based participatory research approach was used to engage migrant women connected to a community-based perinatal program in Edmonton. A focused ethnography was conducted with four Northeast African communities and involved 10 focus groups with women (n = 8, per group) and direct observations of weekly perinatal program activities. Data generation and analysis occurred concurrently, and all generated data were analyzed using qualitative content analysis to inductively derive codes and categories.	A complex network of factors seem to influence Northeast African women’s health during pregnancy and postpartum upon migration to Canada. It is of the utmost importance to provide these women with the immediate sociocultural and environmental factors they need to successfully thrive during pregnancy and postpartum, especially while establishing social and support networks “here”.
Rao VS, *et al.,* / 2020 / Australia.^31^	To explore the experiences of motherhood and postpartum support of Indian migrant mothers.	Indian migrant women’s experiences of motherhood and postnatal support in Australia: A qualitative study.	A Qualitative descriptive natural is inquiry was adopted, with data collected through face-to-face, semi-structured, in depth interviews with a purposive sample of 11 English speaking Indian migrant women over 18 years old, (6 weeks to 6 months postpartum) in 2016. The data were thematically analyzed.	This study gives a unique insight into the experiences of Indian migrant women following birth. There is a need for culturally sensitive and appropriate postnatal services that encourage Indian men to support their partners and help women to find alternative sources of culturally appropriate support. It is vital that mental health support is a key component of any such program of care.

In Table 2, the coping strategies found in the literature are shown and categorized through content analysis into five coping strategies for adaptation that were related to the strategies proposed in Callista Roy's middle-range theory of adaptation to life events. The analysis highlights that the main challenge the articles mention is “maintaining health in pregnancy during migration,” which becomes the main health challenge. This content analysis shows that by using the migrant's adaptation strategies during pregnancy, anxiety and fear are reduced and individualized attention and a positive attitude to life and health are promoted. ^32^

**Table 2:** Coping strategies for adaptation during pregnancy found in the literature, categorized through content analysis related to the strategies proposed by Callista Roy. Health Challenge: Maintain pregnancy health during migration

Adaptation strategies identified in the literature	Categorization of strategies according to content analysis	Callista Roy's Coping Strategy of Adaptation to Life Events
1. Patience in the face of waiting, laughter in the face of pain and gratitude in the face of a right.^15^	1. Strategies for emotional regulation and transfer for maternal health	Duty to be strong
2. a. Search for meaningful relationships with the culture of origin, with the people around them, and the effort to find information with the people around them and/or through books and the Internet. b. Continuous negotiation with themselves and reconstruction of their new identities. c. integration to the new reality.^16^	2. Cultural adaptation strategy: Connections to herself, her ancestors and the future	Bonding and unique connections
3. Cultural adaptation, social support, communication with professional health care personnel. ^17^	3. Comprehensible language strategy: A constant two-way challenge	Common language
4. a. Feelings of being welcomed and treated as an equal. b. A good relationship with the midwife. c. Good communication despite language difficulties and cultural differences.^18^		
5. Positive attitude towards motherhood.^19^	4. Positive attitude strategy to promote maternal health	Focus on the good
6. a. Be open to new opportunities. b. Balancing the sense of belonging. c. Maintaining the original traditions and at the same time be willing to integrate into society. d. Seeking information and support from family and health professionals.^20^		
7. Positive interventions towards motherhood and social support.^21^	5. Social support strategy: A predictor of maternal health	Count on others
8. Family structure and perceived support and how spirituality is integrated.^22^		
9. Social networks.^23^^-^^25^		
10. Multidimensional models of social support.^26^		
11. Maintenance of transnational bonds with family members.^27^		
12. Family financial and emotional support.^28^		
13. Social support and communication with maternal grandparents.^29^		
14. Social support.^30^^,^^31^

Strategies for emotional regulation and transfer for maternal health. This strategy begins with the psychological and physical structuring of the woman and from the attitude towards motherhood that she has during the migration process; that is, how the MPW carry out empowerment, exercise leadership, self-control, improve self-esteem, and fight for their future despite adversity.^25^ When carrying out the analysis of the literature, it was identified that when the MPW, during the migratory process, are left alone and want to continue, they feel the need and the impulse to move forward to improve their health and that of their children.^33^^,^^34^ Thus, being pregnant, they can present different psychological alterations, such as depression, anxiety and fear, which can be exacerbated by the migration process.^25^ If the migrant woman has emotional stability prior to pregnancy, she will better manage the changes she may face. If a woman previously presents psychological disorders, a complicated pregnancy, stressful life circumstances, she will face emotional imbalances during pregnancy such as anxiety and depression, which will lead to a deficit in maintaining her health.^15^

Cultural adaptation strategy: Connections with herself, her ancestors and the future. The articles reviewed indicate that the MPW’s traditions, habits, and cultural practices are still present in the migration process and are presented and shared with the people with whom they interact in the place of transit and/or destination. These connections with culture are an adaptation strategy and a challenge to be explored by the person who cares for them. According to the evidence, this strategy is usually the most complex to carry out by the community receiving migrants but the most important in the process of integration into a territory, a language, educational opportunities, professional training and economic independence, which allows MPW to ensure a future for themselves, their unborn baby, their family, and achieve a reality consistent with their experience.^35^^,^^36^

Comprehensible language strategy: A constant two-way challenge. The challenge that the pregnant woman faces when trying to be understood in the new reality in which she lives occurs in two situations: the first comes from her reality and projection to the environment, and the second from the communication and relationship with others when aiming to receive support and improve the health situation and the bond with her unborn child. In the reviewed literature, the MPW's constant search for a comprehensible language to improve her health, reduce anxiety and fear, through communication with the people who care for her is highlighted.^17^^,^^18^ One of the ways in which the migrant pregnant woman makes her situation known is by making herself visible, another is by achieving understandable communication with the person who cares for her, and the last is by making her culture and customs in health care manifest for her reality to be understood.^(37, 38)^ In the last aspect, the literature considers it relevant that the health professional who cares for the MPW has knowledge about their beliefs, customs, rituals, traditions, and provides comprehensive and individualized care.^18^

Positive attitude strategy to promote maternal health. The most effective strategy to cope with the adversities of the life events or health challenges that arise during the migration process for the MPW is to concentrate and use selective attention to create a positive attitude towards pregnancy, life and health to carry a healthy pregnancy.^(26, 39,40)^ In general terms, the support provided in preparation courses for motherhood and fatherhood, attendance at prenatal check-ups, accompaniment and follow-up of indications, timely access to health services, and motivation to carry out activities pleasant for the migrant pregnant woman could reduce the negative stimulus triggered by the problems associated with migration and thus improve health conditions, promoting the well-being of the maternal-fetal relationship.^19^^,^^20^

Social support strategy: A predictor of maternal health. This strategy will have an effect on the outcome of pregnancy depending on the strength with which it occurs, that is, the greater the social support, the better the outcome of the pregnancy and vice versa.^28^^-^^30^ This strategy is developed when it is evident in the literature that one of the factors generating the most effects during the gestation of MPW is anxiety about loneliness, which manifests itself with emotional and muscular tension, easy crying, startle, trembling; thus, it constitutes a negative factor influencing health status.^21^^,^^28^ It is also evident that MPW with adequate family support in their destination express greater satisfaction with the way they and their family share time, space or money, which generates a positive strategy for adaptation.^22^ According to the reviewed articles; although the support of others is declared, it is not always perceived by the migrant pregnant woman; therefore, the MPW network could be made up of relatives, friends and social networks, which reduce anxiety and fear. It was found that the main source of emotional support for MPW are people not related to their family.^41^

## Discussion

The articles review shows the coping strategies for the adaptation of MPW with respect to their health. These strategies are related and applied to those proposed by Callista Roy, in the Middle-Range Theory of Adaptation to Life Events, taking into account that migration, as a life event, when presented with pregnancy, becomes a health challenge ^13^

Social support is the strategy most evidenced in literature, which is related to the strategy of counting on others proposed by Roy.^42^^,^^43^ The two strategies are intended to demonstrate the importance of support networks to generate a safe and quality pregnancy, which is consistent with studies carried out in Asia on the experiences of migrant women, which found that the adaptation of migrants was carried out through the development of social networks, strong bonds with non-family groups in receiving countries, and the maintenance of transnational ties with relatives from the country of origin and the partner, given that if a MPW has a weak family and marital relationship, this becomes a predictor of anxiety, depression and other mental health problems during pregnancy. ^27^^,^^47^ The social support available to the migrant pregnant woman can be a predictor for the positive outcome of pregnancy, so it is important to characterize them in the transit and destination territories as a support strategy for maintaining her health during pregnancy.^(44, 45)^

Comprehensive language, as a strategy, relates to common language and the unique union and connections found in Callista Roy's middle-range theory.^13^ These strategies seek to generate an alert for the staff and institutions that provide care in the search for an understanding, empathetic, humanized, and inclusive relationship with the MPW.^46^ To confirm the above, a review of qualitative synthesis carried out in 2018 evidences that the access and use of health care for the pregnant migrant was hindered by structural, organizational, social, personal, and cultural barriers of the health institution; care experiences included negative communication, discrimination, poor relationships with health professionals, cultural shocks, and negative experiences of clinical intervention, which generated anxiety and fear.^48^ A study carried out in South America indicates that the practices carried out by migrant pregnant women are rooted in their beliefs, myths, and cultural values inherited from generation to generation, which must be identified by health personnel to offer culturally congruent language and care. ^49^ In a meta-synthesis carried out on interculturality in pregnant migrant women, it is confirmed that the barriers to care fluctuate within the divergence of concepts, little credibility, lack of knowledge of capabilities and limitations, as well as negative experiences in caring for MPW.^50^ In conclusion, it is important to generate intercultural care skills in the people who care for the migrant population that can generate a comprehensive language that involves aspects of humanization and empathy towards the vulnerable population.^(51, 52)^

The emotional regulation and transfer strategy found in the literature is related to the coping strategy proposed by Roy. Being strong during the physical and psychological changes of pregnancy, generates a potential risk for women, which in combination with those of migration, could trigger a deficit in maternal well-being.^53^^,^^54^ When migration involves too many challenges, the most effective strategy that MPW applies is emotional regulation and transfer, which, combined with a positive attitude toward motherhood, facilitates health maintenance.^55^^,^^56^ This coincides with a study carried out in 2017, in which the migration status has a negative effect on perinatal mental health for those who have a poor family support, who do not have a job, are in a precarious immigration situation and/or relationship conflicts; however, when they present an empowerment of their role, personal strengths, family and community emotional resources they have the strength to resist and continue maintaining their health during pregnancy. ^57^ Another study reported in North America indicated how pregnant Mexican migrants had a positive outcome of their pregnancy despite not obtaining prenatal medical care. MPW were guided by beliefs, values, and traditions about self-care during pregnancy related to what to eat, what not to eat, how much exercise and sleep to get, how to avoid stress by exercising, sleeping, and how to stay healthy during pregnancy. They turned to Mexican cultural traditions of care during pregnancy, which allowed them a safe birth.^58^ Consequently, being strong could represent a state of survival for the migrant pregnant woman and her baby, thus it is considered an important pillar to address it from the motivation and work in health and community networks.^(59 60)^

Conclusion. According to the literature reviewed and its connection with Roy's Middle-Range Theory of Life Events, the coping strategy most commonly used by pregnant migrant women to preserve their health and support their adaptation during pregnancy is social support. This includes establishing positive relationships with others, as well as forming or participating in support networks in their countries of origin, transit, and destination. The "being strong" strategy reflects a state of survival for both pregnant migrant women and their children, constituting an essential pillar within community support networks and health systems.

The positive behaviors identified in the literature are oriented toward problem-solving and the mobilization of knowledge, skills, and emotional resources that allow them to maintain a positive attitude toward life and health, contributing to the reduction of mental health disorders. Likewise, the use of understanding language by healthcare personnel, working with migrant women in the coping process, reduces anxiety and fear. In this sense, the cultural competence that professionals must develop when faced with migration phenomena is essential to guarantee equitable, high-quality, and humane maternal care.

Finally, connecting with themselves, their ancestors, and the future enables pregnant migrant women to maintain their overall health and build a new reality in their host territories.

Recommendations: This review recommends that public policies and maternal and perinatal health programs, as well as community health programs, recognize the need and importance of creating social support groups in transit and destination countries to welcome, accompany, and support pregnant migrant women in their processes of change and adaptation, creating inclusive, individualized, and culturally sensitive spaces.

Implications for practice: This review will help professionals and health systems understand the situation experienced by pregnant migrant women, as it invites reflection on the importance of implementing care protocols tailored to their needs and developing research projects aimed at achieving better outcomes during pregnancy, childbirth, and the postpartum period for pregnant women during the migration process.

Limitations of the study. There is little research on the strategies used by migrants to adapt and take care of themselves during pregnancy in a new territory, in addition to the scarce regulations available to support them. The studies found are approached from specific contexts; there is not enough evidence of literature for the care of the pregnant woman, a situation that demands new studies on pregnancy care during migration.
